# Polymer-Assisted Tailings Dewatering in Seawater and Continental Water for Copper Flotation

**DOI:** 10.3390/polym17192613

**Published:** 2025-09-27

**Authors:** Rubén H. Olcay, Andréia B. Henriques, George E. Valadão, Iván A. Reyes, Julio C. Juárez, Martín Reyes, Miguel Pérez, Mizraim U. Flores

**Affiliations:** 1 Departamento de Ingeniería Metalúrgica y Minas, Facultad de Ingeniería y Arquitectura, Universidad Arturo Prat, Avenida Arturo Prat 2120, Iquique 1110939, Chile; 2 Departamento de Ingeniería de Minas, Universidad Federal de Minas Gerais, Belo Horizonte 31270–901, Brazil; 3 Instituto de Metalurgia, Universidad Autónoma de San Luis Potosí, San Luis Potosí 78210, San Luis Potosí, Mexico; 4 Catedrático CONACYT, Consejo Nacional de Ciencia y Tecnología, Benito Juárez 03940, Ciudad de México, Mexico; 5 Área Académica de Ciencias de La Tierra y Materiales, Universidad Autónoma del Estado de Hidalgo, Carretera Pachuca-Tulancingo km 4.5, Carboneras CP 42184, Mineral de la Reforma, Mexico; 6 Área Electromecánica Industrial, Universidad Tecnológica de Tulancingo, Camino a Ahuehuetital, 301, Las Presas, Tulancingo 43600, Hidalgo, Mexico

**Keywords:** dewatering polymer, tailings, water, flotation, thickening, rheology

## Abstract

This study evaluates the use of seawater and continental water in tailings thickening and copper flotation at laboratory scale, focusing on water reuse in mining operations in arid regions. The tailings had a mean particle size of 10 µm, with 75% < 50 µm, and a specific weight of 2.64 g/cm^3^. Seawater contained significantly higher ion concentrations Na^+^ 10,741 ppm, Mg^2+^ 1245 ppm, and Ca^2+^ 556 ppm compared with continental water (187, 32, and 127 ppm, respectively), which negatively affected polymer performance. Sedimentation tests showed that the anionic polymer (A3) increased settling rates by 33 times with continental water at 40 g/t, while with seawater the increase was 31 times at 60 g/t. In column thickener tests, discharge solids reached 65% with continental water and 62% with seawater, representing an annual reduction of ~17,000 m^3^ of recovered water when seawater is used. Consistency tests indicated that achieving slump <20% required 75% solids with continental water and 77.5% with seawater. With dewatering polymers, doses of 200 g/t achieved ~70% solids and slump values near 50%, surpassing column thickener performance. Primary flotation results showed that recirculated and filtered seawater improved copper recovery by 3–5% compared with fresh seawater, due to partial removal of interfering ions. In contrast, recirculated and filtered continental water reduced recovery by 2–4%, likely because of residual polymer effects on mineral surfaces. These findings highlight the importance of polymer selection and dosage optimization to ensure efficient water recovery and sustainable flotation performance under varying water chemistries.

## 1. Introduction

The most important economic activity in Chile is mining, which is concentrated in the northern part of the country. The main mineral products are copper and saline deposits (caliche), composed mainly of natural nitrates. Chile is the world’s leading copper producer, accounting for 24 percent of global production, and, according to the United States Geological Survey (USGS), the second largest producer of lithium, with approximately a 30 percent share of global output. Consequently, millions of tons of minerals are processed every year. The processing of porphyry copper ores generates large volumes of tailings composed of ground rock and slurry from mills, washeries, or concentrators that remain after extracting valuable metals [1,2,3]. In 2020, Chile had 757 tailings deposits, including 112 active, 173 abandoned, 467 inactive, and 5 under construction. In that year alone, active tailings generated 4.337 billion tons [4]. Processing such large quantities of ore requires vast amounts of water, much of which remains trapped in waste dumps. A study conducted in 2015 found that total water consumption by the mining industry was 55.8 m^3^/s, with the Antofagasta region—located in the Atacama Desert—accounting for 51% of national consumption. Mining companies, especially large copper producers, are increasingly seeking to recycle water whenever possible. Mining–metallurgical operations in Antofagasta are particularly water-intensive, consuming about 60% of the region’s freshwater resources [5,6,7,8,9]. The use of seawater in mining is not new; successful applications have been reported in copper, zinc, uranium, and iodine processing. Given the scarcity of freshwater in northern Chile, many companies are now turning to seawater for their operations [10,11]. After desalination, seawater with 3.5 wt.% salinity is pumped to mining plants located more than 1000 m above sea level [12,13,14,15,16,17]. The most water-intensive mining processes worldwide are flotation and leaching [18,19]. Flotation, which separates and concentrates ores based on wettability, involves particle bubble collisions, attachment, and the formation of stable aggregates, making it highly water intensive. As a result, many flotation plants are adopting recycled or seawater as alternatives to freshwater [20,21]. With the depletion of freshwater sources, the use of water with high concentrations of inorganic electrolytes, such as seawater, has become increasingly common in mineral processing [22,23,24,25,26]. Several flotation plants in countries including Australia, Canada, Chile, Indonesia, and Mexico now operate with seawater [27,28,29]. Although seawater poses challenges such as corrosion and process inefficiencies [25,26,30], it also reduces the energy consumption and brine waste associated with desalination [31]. In Chile, both raw and desalinated seawater (via reverse osmosis) are used in mining. A third alternative, partially desalinated seawater, has not yet been adopted. Each option has advantages and disadvantages depending on the ore, the technology, and the project characteristics [32,33,34]. Seawater components may favor, hinder, or have no effect on the recovery and quality of the final product. For existing operations, the use of seawater is practically impossible because facilities are not designed to withstand its corrosive properties. However, raw, desalinated, or partially desalinated seawater can be considered for new projects. In fact, seawater may even improve flotation performance in copper sulfide processing compared with freshwater. The main objective of this study is to evaluate the use of conventional and dewatering polymers in the thickening of copper tailings using both freshwater and seawater, and to assess the reuse of the supernatant in flotation, with a view to improved tailings management and water recycling.

## 2. Materials and Methods

### 2.1. Mineral Sample

**Figure 1:**
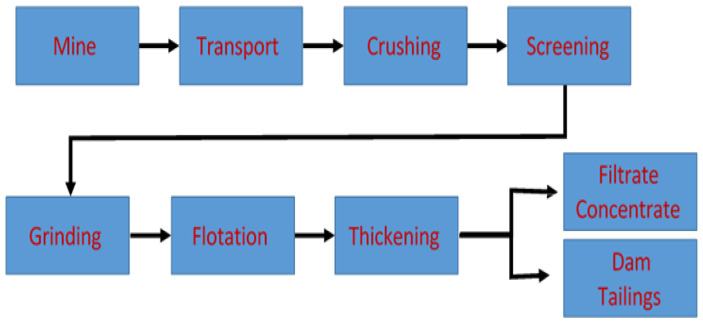
Flow diagram of mineral plant.

The mineral samples used were obtained from final tailings and feed from the copper flotation circuit of a concentration plant located in northern Chile; the flow diagram is shown in Figure 1. The continental water was sourced from groundwater in the Pica community, while the seawater was extracted from the coast of Iquique, both located in the Tarapacá Region in Chile. Table 1 shows the different techniques and equipment used for the physicochemical characterization of the samples and waters.

Table 2 and Table 3 present the main characteristics of the different anionic conventional polymers used in discontinuous sedimentation tests with seawater and continental water.

### 2.2. Discontinuous Tailings Sedimentation Tests with Conventional Polymers

To select the appropriate conventional polymer, discontinuous sedimentation tests were conducted using 1000 mL graduated cylinders. A manual stirrer, operated with ascending and descending movements over a 5 s period for each test, was employed based on methods outlined in previous studies [35,36]. The slurry concentrations were 30% solid mass (M/M), representative of typical final tailings from copper flotation, while the polymer solution concentration was 0.05% (M/V). Both cationic and anionic polymers were evaluated using continental water and seawater. The selection of the conventional polymer was primarily based on the sedimentation rate and the turbidity of the overflow. This polymer was then used in sedimentation tests conducted in a 5000 mL column thickener to obtain the water (overflow) in flotation tests.

### 2.3. Tailings Thickening Tests with Dewatering Polymers

To select the appropriate dewatering polymer, different anionic polyacrylamides were evaluated through consistency tests. For these tests, two 500 mL precipitated glasses were used. In one glass, the polymer dose to be evaluated was prepared at a solution concentration of 0.25% (M/V), as recommended by the manufacturer. In the other, 500 mL of slurry with a solid concentration of 30% (M/M) was placed. The slurry was poured into the glass containing the polymer, and the mixture was transferred back and forth three times. Afterwards, the overflow was collected, and the remaining tailings were added to a PVC cylinder with dimensions of 50 mm in diameter and height, as described in previous studies [35,36,37,38,39].

### 2.4. Tests for the Consistency of the Tailings

Consistency tests were performed using the procedure (NBR NM67, 1998) as determined by Equation (1) where mainly the empty container is filled with the sample with the corresponding percentage of solids in mass for the determination of slump.

(1) $$\%\;Slump=\frac{\left(S\right)}{\left(H\right)}\times 100$$

where *H* is the height cylinder utilized and *S* is height of sample tested.

### 2.5. Tests of Angle of Stacking of the Tailings

**Figure 2:**
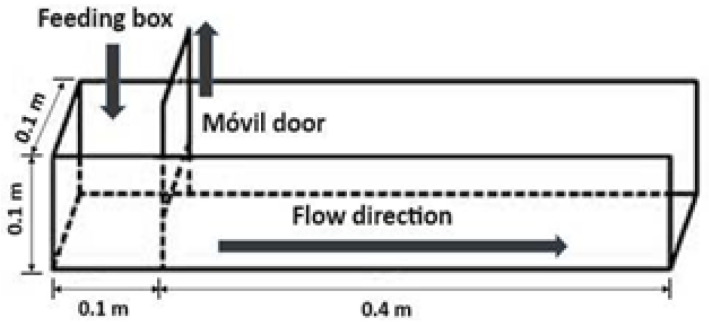
Acrylic equipment.

For the test of angle of stacking of the tailing an acrylic equipment was used (Figure 2). The angles of stacking were obtained by using Equation (2), where h1: initial height of the sample in the equipment, h2: final height of the sample in the equipment, and L: horizontal advance of the sample in the equipment.

(2) $$\theta R=Arctg[\frac{\left(h1-h2\right)}{\left(L\right)}]$$

### 2.6. Yield Stress Measurements in the Tailings

**Figure 3:**
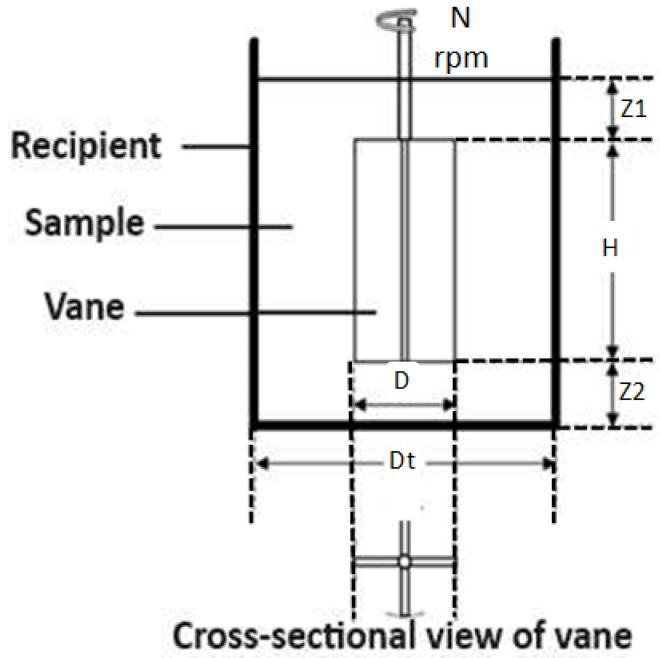
Vane method (modified by Nguyen) and rheometer.

Yield stress tests were performed with different percentages of solids in mass in the Brookfield rheometer model DV3T (Figure 3). In the yield stress tests the vane method was used, where an axis with 4 separate pallets was used every 90°. This was immersed in the slurry sample to a certain depth and the readings were made for a period of approximately 300 s, the turning force was increased to a maximum value, and then gradually decreased until it stopped. The maximum value obtained was used to establish the yield stress using the Bingham model. Figure 3 presents the vane method used, where N is rotation velocity (rpm), D is vane diameter, Dt is diameter of the recipient, H is vane height, and Z is the height of slurry in the bottom and surface.

### 2.7. Tests of Primary Flotation

The primary flotation tests were performed in a “WEMCO” cell with a slurry capacity of 2500 mL. The mineral sample used for laboratory flotation tests comes from feed to the copper flotation circuit of a concentration plant. The main flotation parameters were as follows: feed particle size: 230 µm (P_80_); stirring speed: 900 rpm; pH: 10.5 (lime grout); copper collector dose: 35 g/t, frother dose: 20 g/t; conditioning time: 3 min; rougher flotation time: 7 min; % solid in mass: 32%; airflow rate: 8 L/min; and time between pallet: 15 s.

## 3. Results and Discussion

### 3.1. Characteristics of the Mineral Sample and Water

**Figure 4:**
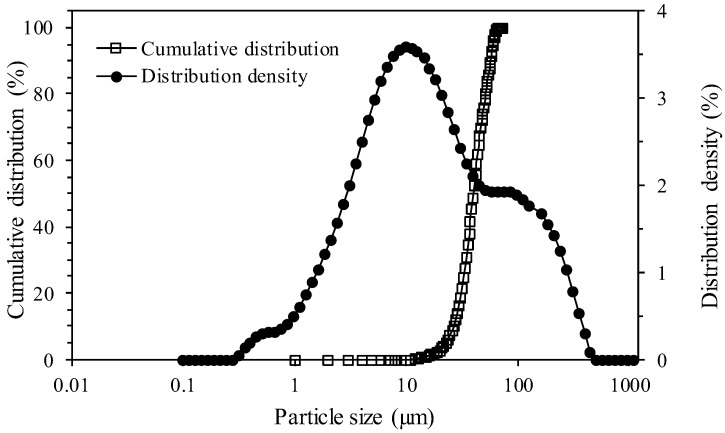
Particle size distribution of the tailings sample.

The specific weight of the final tailings is 2.64 g/cm^3^; the tests were performed in triplicate as shown in Table 4, where the value obtained was used for future calculations. Figure 4 presents the particle size distribution of the tailings sample.

Laser diffraction was used to measure particle size distribution of the fine tailings. Figure 4 shows that the fine tailings have a top size of around 95 µm. The value of the particle size (d_50_) is approximately 10 µm. Tailings are considered fine, because approximately 75% are less than 50 μm (mesh 270). The presence of fine fraction is important for considering the formation of mineral paste and increasing the stability of tailings deposits. It is worth mentioning that the large amounts of fine particles in the tailings, having a larger superficial area, promote physicochemical interactions on their surface with other ions present in the type of water utilized in the process.

According to the results obtained by X-ray fluorescence (Table 5), there are high silicon and iron contents, because the sample has large percentages of quartz and pyrite. The presence of copper is low, so it could be considered that the recovery of copper is high in the beneficiation process. The presence of heavy metals is low, at trace levels, so they are considered difficult to detect by XRD.

**Figure 5:**
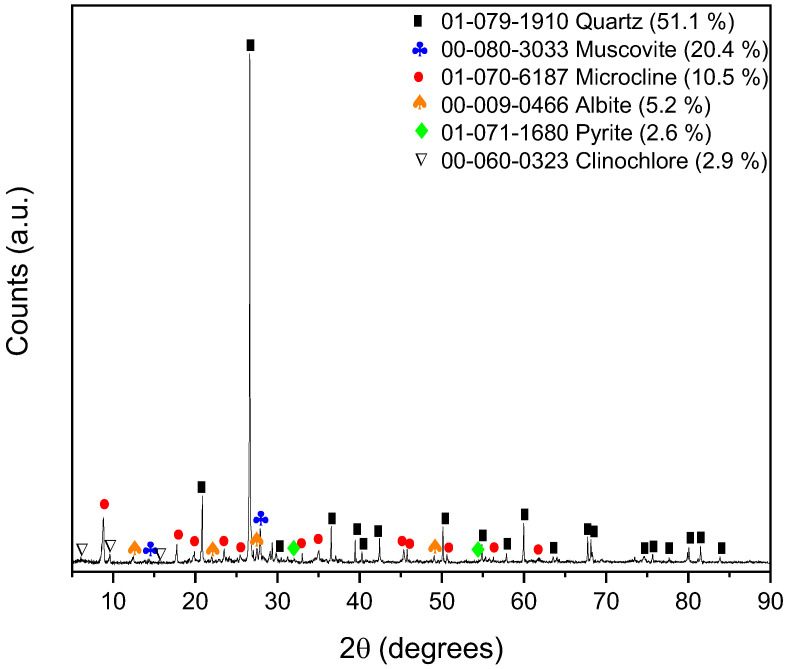
X-ray diffraction of the tailings sample.

Table 6 and Figure 5 show that the mineralogical composition obtained through X-ray diffraction (XRD) analysis reveals a predominance of silicate and aluminosilicate phases, with quartz (SiO_2_), accounting for 55.1% of the total sample. The high content of quartz is indicative of a highly siliceous matrix. The second most abundant mineral phase is muscovite, a mica group phyllosilicate, representing 22.4% of the tailings. The platy morphology of muscovite contributes to the generation of fine particles or slimes during grinding. These slimes are known to adversely affect the flotation process by coating the surface of valuable minerals, hindering collector adsorption, and reducing bubble–particle attachment efficiency. Microcline (KAlSi_3_O_8_) and albite (NaAlSi_3_O_8_) are present in concentrations of 12.4% and 5.2%, respectively. Both minerals belong to the feldspar group and are commonly encountered as gangue in various ore deposits. Their presence may influence the slurry chemistry, particularly the pH and ionic strength of the flotation medium, due to the release of alkali elements. The sample contains minor quantities of pyrite (FeS_2_) and clinochlore, each at 2.6%. Clinochlore, a chlorite group mineral, is another layered silicate which, like muscovite, can negatively affect flotation performance through slime generation and surface contamination of target minerals. It should be noted that the majority of copper tailings in Chile contain elevated levels of quartz.

Table 7 presents the chemical composition of continental and seawater used in the thickening and flotation tests.

The results of Table 5 mainly indicate a large difference between both waters in respect of ions as chlorine and sodium followed by magnesium, calcium, and potassium. Additionally, seawater has a greater electrical conductivity. The high levels of magnesium and calcium present in seawater can interfere with the performance of the copper flotation. This is likely due to the potential formation of magnesium hydroxyl-complexes and for the gypsum precipitation by calcium, which can interfere with the interaction between the mineral and the collector.

### 3.2. Test of Discontinuous Sedimentation

**Figure 6:**
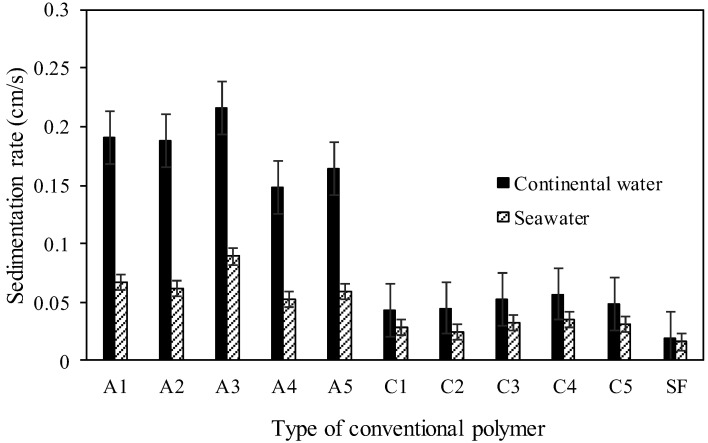
Sedimentation rates of the tailings with continental water and seawater.

Figure 6 shows the sedimentation rates of the tailings with the use of conventional cationic (C) and anionic (A) polymers obtained with continental and seawater; SF is without polymer.

**Figure 7:**
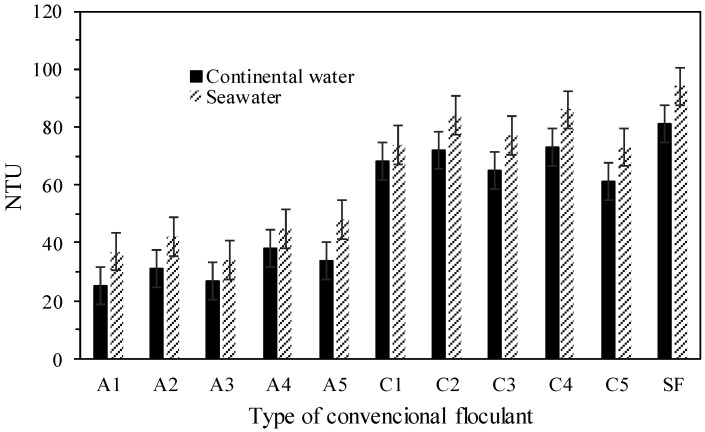
Turbidity of the overflow from discontinuous sedimentation with continental water and seawater.

Figure 6 shows that for all tailings settling rate tests with different conventional polymers, the performance was lower when using seawater. This is likely due to a greater interaction of ions such as calcium, magnesium, chloride, among others present in the seawater, with the surface of the tailings particles and the conventional polymer, thus reducing the settling rate performance. The conventional anionic-type polymers performed better at the sedimentation rate, the polymer named A3 being selected for further testing. It should be noted that the sedimentation rate was increased by approximately 11 and 6 times for continental water and seawater, respectively. Figure 7 shows the results of turbidity of the overflow after 60 min of discontinuous sedimentation.

Figure 7 shows that the turbidity in the supernatant was higher in all tests with seawater, possibly due to the greater interaction between colloidal-sized particles and the ions present in seawater. Despite this, all the conventional polymers evaluated presented a turbidity in the overflow less than 100 NTU, after 60 min of discontinuous sedimentation. It is important to note that pH variations remained below 5% across all tests, with the average slurry pH being 7.8. Turbidity values of supernatant from sources of recuperation such as the thickener, dams, and filters, among others, that are below 100 NTU, are considered highly acceptable for recirculation and reuse in the mineral processing plant.

### 3.3. Discontinuous Sedimentation Tests with Selected Conventional Polymer

**Figure 8:**
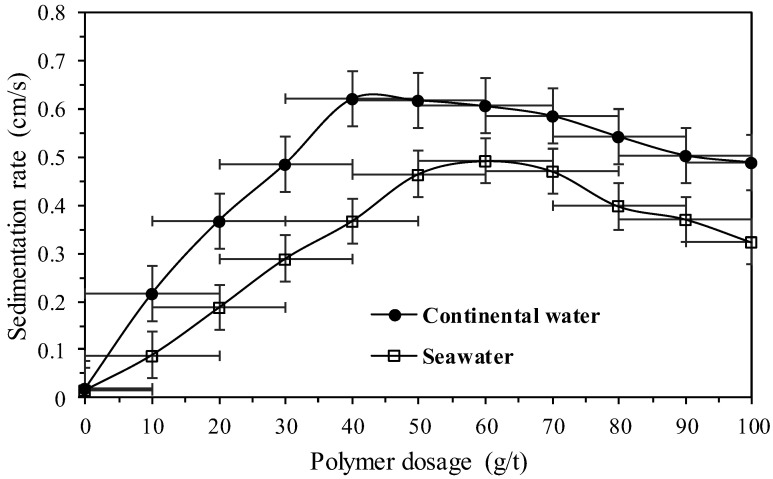
Sedimentation rates for different doses of selected polymer using both continental water and seawater.

Figure 8 presents the assessment of sedimentation rates for different doses of anionic polymer (A3) using both continental water and seawater.

Figure 8 shows that the sedimentation rate is higher when using continental water. It is observed that for a dose of 40 g/t, there is an increase in rate of 33 times, while for seawater with a dose of 60 g/t the rate must be increased by 31 times. In the slurry with continental water a dose of 30 g/t is required to obtain the maximum sedimentation rate using seawater, that is, about half of polymer consumption. Possibly due to the presence of a higher amount of dissolved ions in seawater, such as calcium, magnesium, chloride, potassium, among others, the efficiency of the functional groups of the polymer molecules decreases, which may lead to polymer coiling and lower adsorption onto the mineral particles. In the different conditions of aqueous media, the natural balance between the repulsive and attractive forces between the polymer molecules and the copper tailings surface is crucial in defining the degree of polymer adsorption and, consequently, its operational efficiency.

### 3.4. Discontinuous Sedimentation Tests of Tailings Samples in Column Thickener with Selected Conventional Polymer

**Figure 9:**
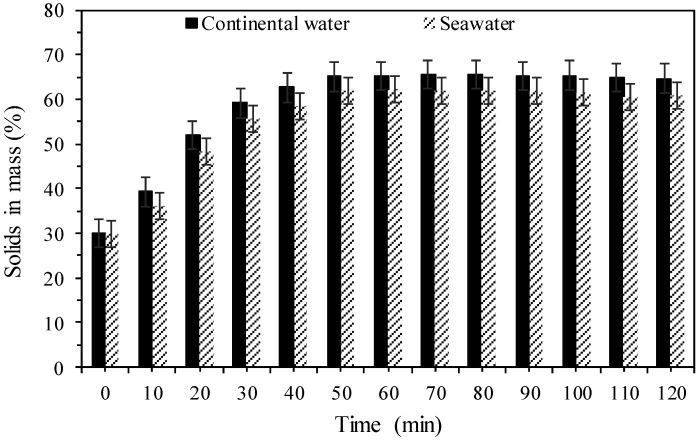
Concentration of solids in mass of the discharge as a function of time with use of continental water and seawater.

Figure 9 shows the evaluation of the percentages of solids in mass of the discharge concentrations of the column thickener for different doses of anionic polymer (A3) using continental and seawater. This shows that the concentration of solids in mass in the discharge reached a maximum in approximately 50 min for both types of water. The concentrations reached were around 65% and 62% for continental water and seawater, respectively, so that both continental water and seawater could be used in this process.

According to the results presented in Figure 9, the solid concentrations obtained in the discharge of the column thickener using seawater were lower compared with continental water, possibly due to lower operational efficiency of the polymer as a result of reduced adsorption on the tailings surfaces. This may lead to lower water recovery in the mineral processing. For example, a thickener discharge of 65% solids in mass instead of 63%, for a slurry feed flow of 100 m^3^/h at 30% solids in mass, means a reduction in directly recovered water from the thickener overflow of approximately 218,920 m^3^ to 201,912 m^3^ per year, that is, an approximate reduction of 17,000 m^3^ of water per year, assuming there are 8000 operational hours annually.

### 3.5. Slurry Stability Without Polymer Use

**Figure 10:**
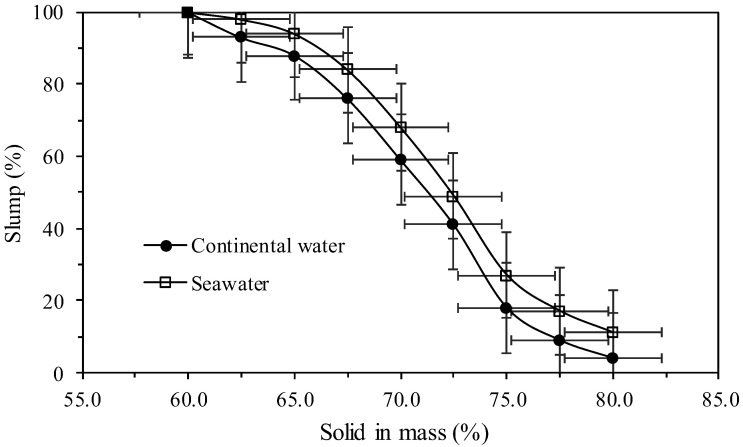
Slump versus solids in mass (%).

Figure 10 presents the evaluation of the consistency of the tailings with different concentrations in percentage of solids in mass with use of continental water and seawater.

Figure 10 shows that the consistency of the tailings changes according to the type of water used, there being in the whole range of solids concentration a greater consistency of the tailings sample with the use of continental water. To obtain a slump of less than 20% a concentration of solids in mass of 75% and 77.5% is required when using continental water and seawater, respectively. That difference in slump is a very important factor in tailings management. For example, it allows for better utilization of the available area in tailings storage facilities, since lower tailings slump enables greater final disposal heights. When using seawater, a 2.5% increase in solids by mass is required to achieve low slump values (less than 20%). This may lead to higher operational and infrastructure costs (Opex and Capex) in mineral processing.

### 3.6. Tailing Consistency Tests Using Dewatering Polymer

**Figure 11:**
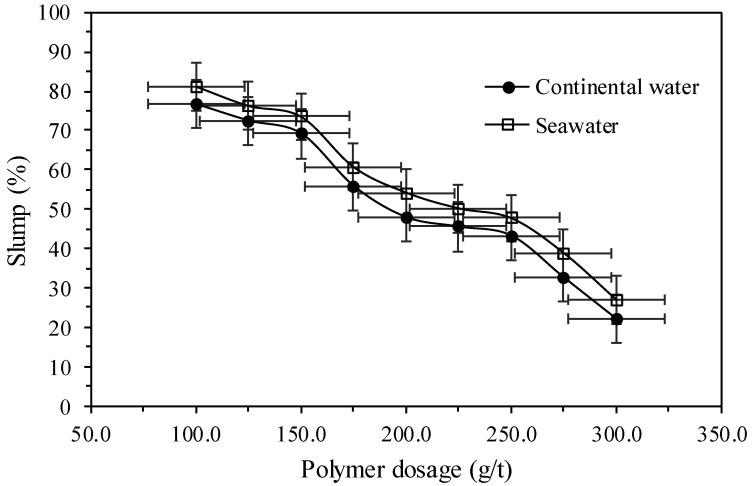
Slump versus dewatering polymer dosage.

The tests of consistency are presented in Figure 11 by means of tailings slump tests with the use of selected dewatering polymer using of continental water and seawater for doses from 100 to 300 g/t, for a slurry concentration equal to 30% of solids in mass.

Figure 11 shows that for all the evaluated doses, slump values were higher when using seawater. In order to achieve slump values below 30% in the tailings, doses around 300 g/t of the dewatering polymer were required. With doses of 200 g/t, solid concentrations of approximately 70% by mass were reached immediately, representing a 40% increase in solids concentration compared to the initial slurry, with slump values around 50% for both types of water. This indicates that the use of dewatering polymer can be considered a viable alternative in the solid–liquid separation stage, as the use of a column thickener resulted in lower solids concentrations than those achieved with the polymer. It is worth noting that in this case, water recovery could be directly reintegrated into the mineral processing circuit, depending on the final tailings disposal method and the supernatant collection system.

### 3.7. Angle of Stacking of the Tailings

**Figure 12:**
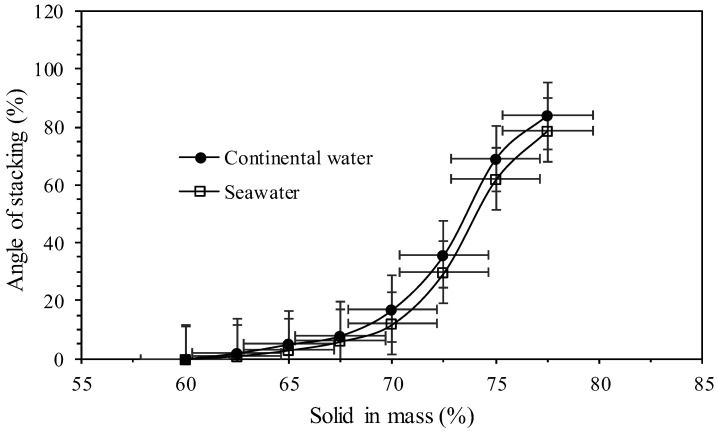
Angles of stacking versus solids in mass (%).

Figure 12 shows the evaluation of the tailings stacking angle with different concentrations in percentage of solids in mass using continental water and seawater.

This parameter is important because knowing the stacking angles of the tailings in advance allows for some level of prediction regarding the tailings disposal capacity in the storage facility. It is expected that higher mass concentrations of tailings will result in steeper stacking angles. However, caution must be taken to respect operational safety factors, such as geotechnical stability, to ensure that the containment structures (dam) are not compromised. Figure 12 shows that all the angles of repose obtained using seawater were lower than those with continental water. Starting at 65% solids in mass in the tailings, an increase in the angle of repose is observed, which may indicate the onset of a change in the slurry rheology. At 65% solids in mass, angles of repose between 3 and 5° were obtained, values that can be considered acceptable for the final disposal of tailings.

### 3.8. Yield Stress of the Tailings

**Figure 13:**
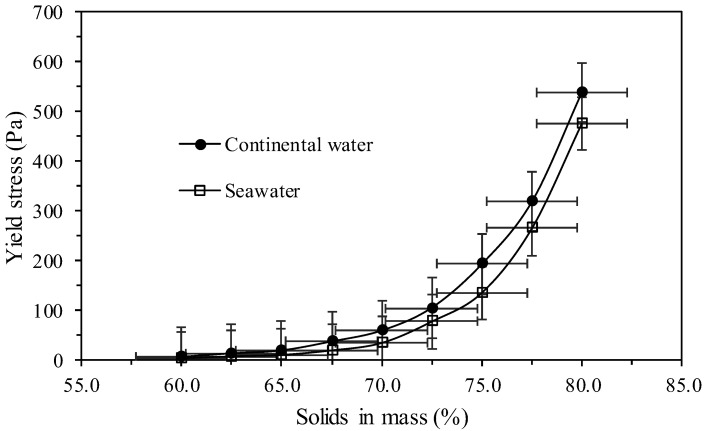
Yield stress versus percentage of solids in mass (w/w).

Figure 13 shows the evaluation of the relative yield stress with different concentrations in percentage of solids in mass, using both continental water and seawater.

This parameter is important in order to determine the equipment required for the tailings transport systems, such as centrifugal pumps, positive displacement pumps, conveyor belts, among others, to the final disposal points in the tailings storage facilities. Figure 13 shows that the yield stress of the tailings changes according to the type of water used, there being in the whole range of concentration of solids in mass a greater yield stress related to the use of continental water. When solid concentrations by mass reach 70%, the tailings are in the form of high-density slurry (values < 100 Pa). For values around 75% (values > 100 Pa) and 80% (values > 450 Pa), the tailings present as paste and cake, respectively.

### 3.9. Analysis of Continental Water and Seawater Used in the Process

Table 8 presents the chemical composition of continental water and seawater used in the flotation tests.

According to the results presented in Table 8, the use of the filtering medium demonstrated a notable adsorption capacity for divalent ions such as calcium (Ca^2+^) and magnesium (Mg^2+^). In the case of seawater, reductions in magnesium ion concentrations of approximately 16% with the conventional polymer and 13% with the dewatering polymer were observed. These results are consistent with previous studies that highlight the effectiveness of various adsorption and precipitation methods for divalent ions in saline waters. For example, Wang et al. (2021) demonstrated that a calcium alginate–sodium citrate composite aerogel could effectively remove Ca^2+^ and Mg^2+^ from water through ion exchange and surface complexation mechanisms [40]. Similarly, Dong et al. (2019) reported significant reductions in calcium and magnesium concentrations in seawater using microbial-induced carbonate precipitation, confirming the potential of targeted treatments to control divalent ion content [41]. In contrast, the sodium ion (Na^+^) exhibited a lower reduction in both seawater and continental water, likely due to its higher solubility and monovalent nature, which hinder its adsorption onto the filtering medium. This behavior aligns with findings by Sharkh et al. (2022), who discussed the challenges of removing monovalent ions such as Na^+^ from seawater during resource recovery and desalination processes, emphasizing the relative difficulty of adsorbing highly soluble monovalent ions compared to divalent ions [42]. Overall, the observed reductions in Ca^2+^, Mg^2+^, and, to a lesser extent, Na^+^ in the supernatants confirm that the filtering medium, in combination with conventional and dewatering polymers, is capable of partially mitigating ionic loads under the tested conditions. These results highlight the practical potential of adsorption-based treatments to manage water chemistry in flotation and mineral processing systems, particularly when dealing with seawater recirculation and water reuse scenarios.

### 3.10. Flotation Tests with Continental Water and Seawater

**Figure 14:**
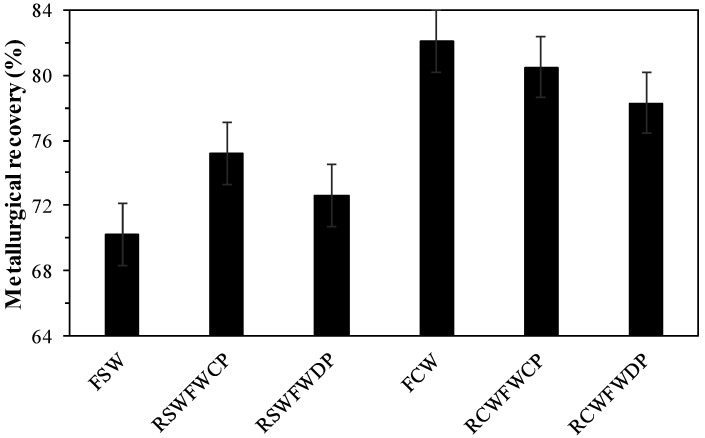
Flotation of copper versus type water.

Figure 14 shows the average results obtained from the primary flotation carried out in triplicate with the use of fresh seawater (FSW), recirculated seawater filtered with conventional polymers (RSWFCP), recirculated seawater filtered with dewatering polymers (RSWFDP), fresh continental water (FCW), recirculated continental water filtered with conventional polymers (RCWFCP), and recirculated continental water filtered with dewatering polymers (RCWFDP).

According to Figure 14, in primary flotation tests it was observed that with the use of recirculated and filtered seawater with application of conventional and dewatering polymers better performances are obtained to the use of fresh water with increases in metallurgical recovery around 5% and 3%, respectively. This could be due to the fact that the concentration of magnesium ions present in seawater was reduced. Under alkaline conditions, high salinity may promote the formation of precipitates on the mineral surface, which can hinder collector adsorption and negatively impact the overall efficiency of the flotation process. In the case of the use of recirculated and filtered continental water with application of conventional and dewatering polymers, it presented a reduction in metallurgical recovery of primary flotation compared to the use of fresh continental water, around 2% and 4%, respectively, probably due to the fact that traces of residual polymers in recovered water may influence the surface properties of copper minerals by modifying their physicochemical surface characteristics. Residual polymers from the recovered water in the mineral dewatering stages that were not degraded mechanically, chemically, or biologically can influence the flotation of copper minerals in various ways, such as having a depressant effect due to repulsive or attractive forces between the residual polymer and the mineral surface of interest. It is therefore crucial to note that during water thickening and recirculation in flotation, some flocculants may remain as residual polymers in the solution, which can impact flotation efficiency by altering collector adsorption, bubble stability, and overall copper recovery [43,44].

## 4. Conclusions

This study provided a comprehensive evaluation of copper tailings dewatering using both continental water and seawater, with emphasis on water reuse strategies for flotation in arid mining regions. Tailings were characterized by a fine particle size distribution (d_50_ ≈ 10 µm; 75% < 50 µm), which increased polymer demand and reduced natural sedimentation rates. Quantitative results confirmed that anionic polymer (A3) substantially enhanced settling performance, achieving a 33-fold rate increase in continental water at 40 g/t and a 31-fold increase in seawater at 60 g/t. Column thickener tests reached underflow concentrations of 65% solids with continental water and 62% with seawater, implying a reduction of ~17,000 m^3^/year in directly recovered water when seawater is used. Consistency tests showed that slump <20% required 75% solids in continental water and 77.5% in seawater. Dewatering polymers provided an additional advantage, as doses of 200 g/t yielded ~70% solids and slump values near 50%, surpassing column thickener performance. In flotation, recirculated and filtered seawater improved copper recovery by 3–5% compared with fresh seawater, while recirculated continental water caused a 2–4% decrease, likely due to residual polymer interactions with mineral surfaces. These outcomes highlight a clear trade-off: seawater requires higher polymer dosages but, when combined with filtration, can deliver both acceptable thickening and superior flotation results. More broadly, the findings emphasize that site-specific water management strategies based on polymer type, dosage, and water treatment are essential to balance water conservation, tailings stability, and metallurgical performance. Such approaches are essential to ensure sustainable mining operations in water scarce regions.

## Figures and Tables

**Table 1:** Techniques and equipment for physics and chemical characterization of the samples and waters.

Property	Technical	Equipment
Tailings specific weight	Simple pycnometry	Pycnometer
Tailings particle size distribution	Laser diffraction	Marveln Mastersizer 3000 (Worcestershire, UK)
Tailings yield stress	Rheometry	Rheometer Brookfield DV3T (United States, as Brookfield Engineering Laboratories)
Tailings chemical composition	X-ray fluorescence	Olympus Delta Premium DP-4050-C (Waltham, MA, USA)
Tailings mineral composition	X-ray diffraction	Advance Davinci Bruker D8 Spectrometer (Bruker AXS GmbH, Karlsruhe, Germany)
Water chemical composition	Atomic absorption spectrometer	SpectrAA-55B (Santa Clara, CA, USA)
Water turbidity	White light source and 90°detection	Turbidimeter TB 250 WL (Lovibond, which, Tintometer Group, Germany)

**Table 2:** Main characteristics of anionic conventional polymer used in discontinuous sedimentation test.

Flocculant/Charge	Molecular Weight	Functional Groups	Degree of Hydrophilicity	Conformation in Solution	Behavior in the Presence of Ions
A1/Anionic	High	Amide + carboxylate	Hydrophilic	Extended	High salinity → partial compaction; Cu^2+^ → dense flocs
A2/Anionic	Very high	Amide + COO^−^	Very hydrophilic	Highly extended coil	Tailings thickeners; SO_4_^2−^ → maintains efficiency up to a certain limit
A3/Anionic	High	Anionic polyacrylamide (amide + carboxylate)	Very hydrophilic	Extended chain; good bridging	With Ca^2+^/Mg^2+^ → more compact flocs; with Cu^2+^/Fe^3+^ → strong interactions, rapid settling
A4/Anionic	Medium	Amide + carboxylate (copolymer)	Hydrophilic	Less extended than APAM	Better tolerance in waters with SO_4_^2−^; with Ca^2+^ → stable flocs
A5/Anionic	High	Amide + sulfonate (–SO_3_^−^)	Very hydrophilic	Coil expanded by sulfonate charges	High performance in recirculated waters with copper sulfates

**Table 3:** Main characteristics of cationic conventional polymer used in discontinuous sedimentation test.

Flocculant/Charge	Molecular Weight	Functional Groups	Degree of Hydrophilicity	Conformation in Solution	Behavior in the Presence of Ions
C1/Cationic	High	Amide + quaternary ammonium	Hydrophilic	Extended chain	SO_4_^2−^ reduces efficiency; Cu^2+^ → compact flocs
C2/Cationic	Medium	Amide + quaternary ammonium	Hydrophilic	More compact	Pre-aid before anionics; good adsorption on fines
C3/Cationic	Low	Protonated amines	Very hydrophilic	Compact, direct neutralization	Resistant to SO_4_^2−^; affinity for negative clays
C4/Cationic	Medium	Protonated branched amines	Hydrophilic	Branched, multiple adsorption sites	Improves initial settling in waters with Cu^2+^/Fe^3+^
C5/Cationic	Medium	Quaternary ammonium	Very hydrophilic	Short chain, surface layer	Little affected by Ca^2+^/Mg^2+^; maintains adsorption in waters with sulfates

**Table 4:** Specific weight of the tailings, calculated by simple pycnometry.

Tailings	Sample I	Sample II	Sample III	Average
Specific weight (g/cm^3^)	2.65	2.59	2.69	2.64

**Table 5:** Chemical composition of the sample made by X-ray fluorescence.

Sample	Si (%)	S (%)	K (%)	Ca (%)	Fe (%)	Cu (%)	Zn (%)	As (%)	Zr (%)	Mo (%)	Pb (%)	LE (%)
**Tailing 1**	**5.23**	**0.94**	**2.03**	**0.64**	**3.54**	**0.11**	**0.020**	**0.02**	**0.01**	**0.04**	**0.01**	**86.11**
**Tailing 2**	**4.46**	**0.88**	**2.01**	**0.61**	**3.89**	**0.13**	**0.010**	**0.02**	**0.01**	**0.05**	**0.01**	**87.44**
**Tailing 3**	**5.07**	**0.89**	**2.1**	**0.54**	**3.7**	**0.11**	**0.02**	**0.02**	**0.01**	**0.01**	**0.01**	**86.53**
**Average**	**5.92**	**0.91**	**2.04**	**0.6**	**3.71**	**0.12**	**0.01**	**0.02**	**0.01**	**0.03**	**0.01**	**86.70**

**Table 6:** Mineral species of the sample made by XRD.

Mineral Species	Formula	Percentage in the Sample
Quartz	SiO_2_	55.1
Muscovite	K_2_(Mg,Al)_4–5_(Al,Si)_8_O_20_(OH)_4_	22.4
Microcline	KAlSi_3_O_8_	12.4
Albite	NaAlSi_3_O_8_	5.2
Pyrite	FeS_2_	2.6
Clinochlore	Mg_3.75_Fe^2+^_1.25_Si_3_Al_2_O_10_(OH)_8_	2.6

**Table 7:** Chemical analysis of collected continental and seawater.

Water Sample	Na (ppm)	Mg (ppm)	Ca (ppm)	pH	EC (σ) (mS/cm)	Turbidity (NTU)
Continental	187	32	127	7.7	1.4	<100
Seawater	10,741	1245	556	7.9	56	<100

**Table 8:** Chemical analysis of recirculated and filtered water samples, using conventional polymer (CP) and dewatering polymer (DP).

Water Sample	Na (ppm)	Mg (ppm)	Ca (ppm)	pH	EC (σ) (mS/cm)
Continental CP	161	25	105	7.8	1.2
Seawater CP	9764	1043	471	8.0	44
Continental DP	172	29	116	7.9	1.1
Seawater DP	9975	1081	514	8.1	49

## Data Availability

The original contributions presented in this study are included in the article. Further inquiries can be directed to the corresponding authors.
